# Unexpected Information Demand and Volatility Clustering of Chinese Stock Returns: Evidence from Baidu Index

**DOI:** 10.3390/e22010044

**Published:** 2019-12-28

**Authors:** Gang Chu, Xiao Li, Dehua Shen, Yongjie Zhang

**Affiliations:** 1 College of Management and Economics, Tianjin University, Tianjin 300072, China; chugang@tju.edu.cn (G.C.); dhs@tju.edu.cn (D.S.); yjz@tju.edu.cn (Y.Z.); 2 School of Finance, Nankai University, Tianjin 300350, China

**Keywords:** volatility clustering, Baidu Index, information demand, generalized autoregressive conditional heteroscedasticity model (GARCH), mixture of distribution hypothesis

## Abstract

This paper employs the Baidu Index as the novel proxy for unexpected information demand and shows that this novel proxy can explain the volatility clustering of Chinese stock returns. Generally speaking, these findings suggest that investors in China could take advantage of the Baidu Index to obtain information and then improve their investment decision.

## 1. Introduction

Recently, scholars have begun to employ Internet news as the proxy for information flow to explain the volatility clustering of stock returns. For example, Zhang et al. firstly employed the number of news which appeared in Baidu News as the novel proxy for information arrival and showed that this proxy could explain the volatility clustering of the SME PRICE INDEX [1]. Based on the Mixture of Distribution Hypothesis (MDH), Shen et.al. [2] further showed that this novel proxy could also explain the volatility clustering for individual stocks. This Internet news-based proxy has gained increasing popularity and is used in various empirical studies, such as in [3,4,5,6], among others. However, the key drawback of this Internet news-based proxy is that media outlets may not play a role in diffusing information, and the observed phenomenon, such as reduced volatility clustering, is driven by investor sentiment or psychological biases [4,7,8]. In this paper, we construct a novel proxy for unexpected information demand based on the search frequency of the Baidu Index, and show that this novel proxy could explain the volatility clustering of stock returns in the Chinese stock market.

In that sense, the contribution of this paper is twofold. Firstly, unlike prevailing studies employing trading volume as the proxy for information flow [9,10,11,12,13], we advocate a novel proxy by calculating the unexpected information demand with the Baidu Index. The rationale to employ the Baidu Index is that: Zhang et al. [14] claims that compared with Google Trends, the Baidu Index provides more scientific, authentic, and objective data, and the search results are given at daily frequency. In particular, our results show that the Baidu Index explains more volatility clustering compared to the studies relying on trading volume as the proxy for information flow, such as those by [10] and [12]. Secondly, we provide stock-level evidence that Internet information could explain volatility clustering by focusing on 40 stocks in the Chinese stock market. To the best of our knowledge, we are the first to employ the Baidu Index to explain the volatility clustering at stock level.

The remainder of this paper is structured as follows: Section 2 describes the data and variables construction; Section 3 gives the research methodology; Section 4 gives the empirical results; and Section 5 presents the conclusions.

## 2. Data and Variables Construction

We used the daily stock closing price over the whole period of 1 January 2015 to 31 December 2018 from the China Stock Market & Accounting Research Database (CSMAR). The following model was used to calculate the daily return of stocks:

(1) $$Return_{i,t}=\frac{\left(ClosingPrice_{i,t}-ClosingPrice_{i,t-1}\right)}{ClosingPrice_{i,t-1}}$$

where the $Return_{i,t}$ represents the return of stock i on day t, and the $ClosingPrice_{i,t}$ represents the closing price of stock i on day t.

In this paper, we used keyword search volume data from the Baidu Index instead of Google Trend. The Baidu search engine is the biggest search engine in China, and we collected the search volume time series data from the website (https://index.baidu.com). The abnormal change of the Baidu search volume represents the unexpected information demand. We followed Drake, Roulstone, and Thornock [15] to define the abnormal search volume:

(2) $${\bar{BSVI}}_{i,t}=\frac{1}{10}\overset{10}{\underset{k=1}{\sum }}BSVI_{i,t-7\;\times \;k}$$

(3) $$AbSearch_{i,t}=\frac{BSVI_{i,t}-{\bar{BSVI}}_{i,t}}{{\bar{BSVI}}_{i,t}}$$

where, $BSVI_{i,t}$ represents the Baidu search volume of stock i on day t. $AbSearch_{i,t}$ represents the abnormal search volume of stock i on day t. We defined $AbSearch_{i,t}$ as that Baidu search volume (BSVI) on day t for stock i less the average BSVI for the same stock and weekday over the previous 10 weeks, and divided it by the average BSVI for the same stock and weekday over the previous 10 weeks.

**Figure 1:**
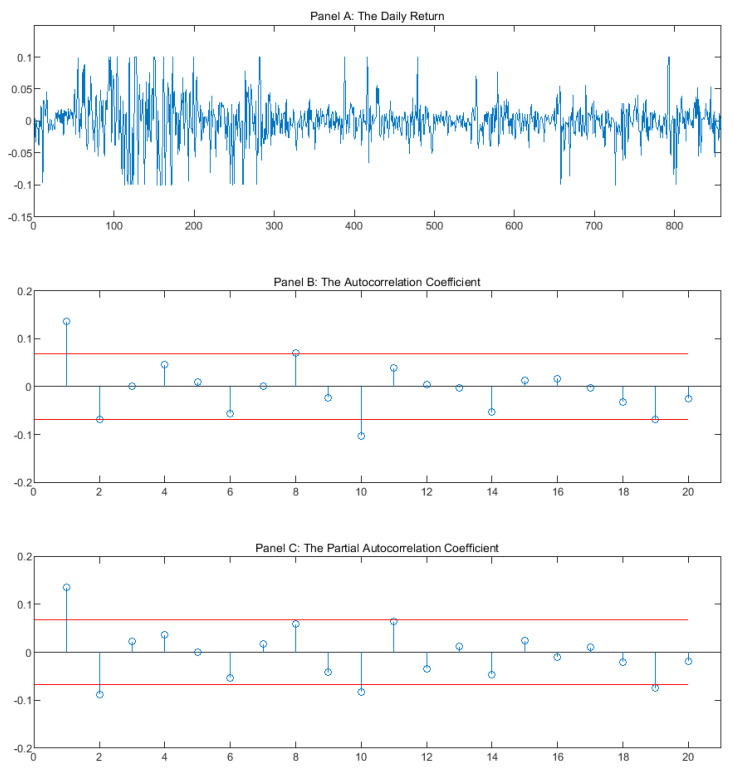
This figure shows the daily return, the autocorrelation coefficient, and the partial correlation coefficient of SHENZHEN ZHENYE(GROUP) CO., LTD (000006.SZ).

We randomly selected 40 stocks from the whole stock market which have a significant autoregressive conditional heteroscedasticity model (ARCH) effect. Figure 1 illustrates the daily return, the autocorrelation coefficient, and the partial correlation coefficient of SHENZHEN ZHENYE (GROUP) CO., LTD (Shenzhen, China) (000006.SZ). We found that the autocorrelation coefficient and the partial correlation coefficient are significantly different from zero (the value exceeds the confidence level), which show that the return time-series of stock 000006.SZ has a significant correlation.

To examine the ARCH effect in residuals, we used two different tests—The Ljung-Box-Pierce Q squared residual correlation diagram and the ARCH Lagrange Multiplier (LM) test. We used Ljung-Box-Pierce Q to investigate the autocorrelation and partial correlation for the squared residuals of the mean equation. Table 1 reports that the Ljung-Box-Pierce Q test is statistically significant at the 5% level in 5-order, 10-order, 15-order, and 20-order lags for the 40 stocks. It denotes that there is significant autocorrelation for all 40 stocks and indicates a significant ARCH effect in the residuals of the mean equation.

The ARCH Lagrange Multiplier LM test was calculated by an auxiliary test regression and used to test the heteroscedasticity of the time-series. Table 2 reports that the LM values are statistically significant at the 5% level in 5-order, 10-order, 15-order, and 20-order lags for the 40 stocks. It indicates the existence of an ARCH effect in the residuals sequence. Hence, the Generalized ARCH (GARCH) model is appropriate to use for all the 40 stocks.

The results of the Ljung-Box-Pierce Q and ARCH Lagrange Multiplier (LM) tests show that there is serious heteroscedasticity and autocorrelation on returns of the stock, and the GARCH(1,1) model fits the data well. We used GARCH(1,1) to calculate the daily return volatility. The GARCH(1,1) model is as follows:

(4) $$\varepsilon_{t}=\sqrt{h_{t}}\nu_{t}$$

(5) $$h_{t}=\alpha_{0}+\beta_{1}h_{t-1}+\alpha_{1}\varepsilon_{t-1}^{2}$$

Table 3 reports the Pearson and Spearman correlation coefficients between daily return volatility and the Baidu search volume. This table suggests that there is positive significant contemporaneous correlation between daily return volatility and Baidu search volume in all 40 stocks. Furthermore, the mean of the Pearson correlation coefficients is 0.6428, and the mean of the Spearman correlation is 0.6398, which denote that these two variables are highly correlated.

To further consider the relation between the daily return volatility and Baidu search volume, we introduced another direct measure, namely, mutual information. Mutual information is a useful indicator in information theory to measure relative information, and it is widely used to measure the correlation between two different variables. To measure the correlation between two equal length time series $\left\{x_{t}\right\}$ and $\left\{y_{t}\right\}$, t=1, 2, 3,…, N, we computed the mutual information between these two time series, as follows:

(6) $$MI\left(X,Y\right)=\int_{Y}^{}\int_{X}^{}p\left(x,y\right)\log\left(\frac{p\left(x,y\right)}{p\left(x\right)p\left(y\right)}\right)dxdy$$

where p(x,y) is the joint probability density distribution function of X and Y; p(x) is the marginal probability density distribution function of X, and p(y) is the marginal probability density distribution function of Y.

Table 4 represents the mutual information between the daily return volatility and abnormal Baidu search volume. All 40 stocks showed a positive value of mutual information, and the mean of mutual information is 0.7106, which denotes that these two variables are highly correlated. The empirical results clearly support that there is a significant correlation between the Baidu index and daily return volatility.

## 3. Methodology

In the time series financial model, the disturbance variance is often found to be less stable. The conditional variance of the error term usually varies with time and relies on the magnitude of the previous errors. In order to solve the heteroscedasticity issue, Bollerslev [16] proposed the generalized autoregressive conditional heteroscedasticity model (GARCH), which is designed to deal with the volatility persistence and describe how the amplitude of return varies over time. In this paper, the GARCH(1,1) model was adopted due to the fact that it has been shown to be suitable to deal with conditional variance that fits many financial time series quite well [16,17]. The GARCH model can be described by the following models:

(7) $$R_{t}=\mu +\varepsilon_{t},\;where\;\varepsilon_{t}|\Omega_{\;t-1}\sim \;\left(0,h_{t}^{2}\right)$$

(8) $$h_{t}^{2}=\omega +\alpha \varepsilon_{t-1}^{2}+\beta h_{t-1}^{2}$$

where $R_{t}$ represents the stock return at day t. μ is a constant, $\varepsilon_{t}$ represents the serially uncorrelated errors, and $h_{t}^{2}$ represents the conditional variance of the $\varepsilon_{t}$. The sum of the coefficients α and β indicates the degree of volatility persistence.

The Baidu search volume index (SVI) is an ideal proxy for information demand because this variable reflects effort by the investor to obtain firm-specific financial information. The abnormal search volume (AbSearch) represents investors’ demand to search for information. Clark [18] proposed the Mixture of Distributions Hypothesis (MDH), and believes that the price time varying conditional volatility is associated with the information flow. According to the MDH, we made a rational assumption that introducing a proxy of information arrival into the variance model will decrease the observed volatility clustering. Therefore, we proposed an extended model that contains an abnormal Baidu search volume, which can be written as follows:

(9) $$h_{t}^{2}=\omega +\alpha \varepsilon_{t-1}^{2}+\beta h_{t-1}^{2}+\lambda AbSearch_{t}$$

If the assumption is correct, the volatility persistence, represented by α+β, should be significantly reduced in comparison with the benchmark model, that is, the original GARCH(1,1) model.

## 4. Empirical Results

**Figure 2:**
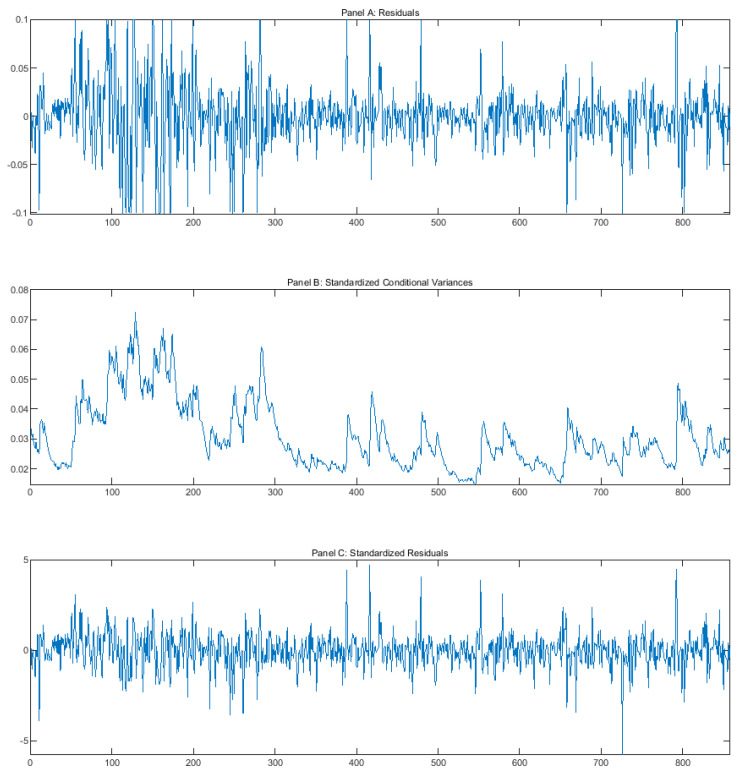
This figure shows the residuals, standardized conditional variance, and standardized residuals of SHENZHEN ZHENYE(GROUP) CO., LTD (000006.SZ).

We firstly focus on the estimation results of the benchmark GARCH(1,1) model. In an unreported table, both the coefficients α and β are statistically significant at the 1% level. The sum of the coefficients α + β range from 0.998455 to 0.769458 with a mean value of 0.904028. Figure 2 illustrates the residuals, standardized conditional variance, and standardized residuals of SHENZHEN ZHENYE (GROUP) CO., LTD (000006.SZ). We find that the benchmark model fits the volatility dynamic quite well. Table 5 presents the estimation results of the extended model that contains AbSearch. All the coefficients α, β, and γ of the extended GARCH(1,1) model are statistically significant at the 1% level. The sum of the coefficients α + β range from 0.87521 to 0.489454 with a mean value of 0.698305. Table 6 reports the summarized results for the degree of volatility clustering, indicating that α + β is significantly decreased. In particular, we found that after incorporating the proxy for the unexpected information demand, the sum of the coefficients α + β dropped significantly with an average of 0.205723. All these findings suggest that the GARCH(1,1) model captures the volatility clustering well, and the unexpected information demand was able to explain the volatility clustering.

## 5. Conclusions

This paper employed the Baidu search volume index (BSVI) as the novel proxy for unexpected information demand and validates the MDH. BSVI represents investors’ searching behavior through the channel of Baidu, which is the largest search engine in China. In that sense, BSVI is a suitable proxy for the information demand. To test the contemporaneous correlation, we employed the Pearson and Spearman correlation coefficients, as well as the mutual information between BSVI and returns and volatiles. The empirical results based on the GARCH(1,1) model reveal a positive and significant impact of the abnormal Baidu Search volume on the conditional volatility of stock return. Generally speaking, these findings suggest that investors in China could take advantage of the Baidu Index to gather information about the stock market and then improve their financial decision-making. For example, investors could employ the high-frequency news to “nowcast” the return volatility, and thus make the optimal investment decision.

## Figures and Tables

**Table 1:** The results of Ljung-Box-Pierce Q.

Stock Code	5-Lags	10-Lags	15-Lags	20-Lags
000006.SZ	21.6051 ***	38.4482 ***	42.4545 ***	48.2823 ***
000009.SZ	8.0681	20.2608 **	50.1010 ***	57.5135 ***
000012.SZ	5.9939 ***	22.6637 **	30.2325 **	39.3211 ***
000014.SZ	24.7229 ***	39.5542 ***	60.2213 ***	68.8523 ***
000032.SZ	18.5736 ***	35.9589 ***	41.3653 ***	55.0598 ***
000055.SZ	16.1696 ***	25.1449 ***	35.4292 ***	43.4041 ***
000058.SZ	20.4609 ***	31.4845 ***	36.7648 ***	42.5812 ***
000060.SZ	13.4690 **	14.2572	32.2536 ***	33.5518 **
000062.SZ	24.7848 ***	28.1216 ***	36.2232 ***	50.3635 ***
000063.SZ	15.2538 ***	25.4355 ***	28.4957 **	33.0139 **
000070.SZ	30.8077 ***	46.3809 ***	49.2339 ***	52.8709 ***
000088.SZ	22.7924 ***	52.1273 ***	56.3863 ***	65.1081 ***
000417.SZ	5.0400	26.7173 ***	40.2973 ***	43.4603 ***
000151.SZ	18.5001 ***	43.9111 ***	61.21 ***	63.2111 ***
000428.SZ	18.8814 ***	39.0454 ***	54.2291 ***	58.0627 ***
000488.SZ	10.6168 *	21.6755 **	31.5148 **	42.4101 ***
000501.SZ	10.5518 *	43.9884 ***	59.9645 ***	72.0285 ***
000506.SZ	83.1071 ***	96.4226 ***	109.1136 ***	110.5655 ***
000572.SZ	2.5366	25.0778 ***	28.9072 **	38.4491 ***
000544.SZ	16.2461 ***	28.9105 ***	39.0724 ***	52.2869 ***
000545.SZ	15.6275 ***	20.2738 **	30.3052 **	49.6051 ***
000548.SZ	7.1463	28.3473 ***	42.6825 ***	49.8242 ***
000551.SZ	10.6251 *	22.0099 **	29.1647 **	31.4479 **
000552.SZ	9.4039 *	11.0429	39.3816 **	42.3156 ***
000554.SZ	10.2074 *	14.6459	19.2328	21.6216
000559.SZ	11.7471 **	35.2579 ***	40.7503 ***	42.0554 ***
000561.SZ	13.8186 **	18.5989 **	29.6349	39.4647 ***
000565.SZ	16.7915 ***	19.1473 **	30.4736 **	37.2379 **
000570.SZ	16.6161 ***	33.7508 ***	52.1117 ***	58.3116 ***
000576.SZ	61.1017 ***	68.8473 ***	72.7982 ***	89.2181 ***
000597.SZ	6.4010	21.4204 **	31.0142 **	34.1832 **
000600.SZ	9.1283 *	28.6727 ***	37.5731 ***	48.2757 ***
000603.SZ	24.6331 ***	36.1251 ***	47.0366 ***	68.8977 ***
000619.SZ	23.0051 ***	34.4507 ***	47.1044 ***	49.5425 ***
000628.SZ	18.5710 ***	23.5099 ***	27.5565 **	35.9639 ***
000629.SZ	12.6282 **	17.6407 **	20.1427	30.2449 *
000631.SZ	5.3062	21.3837 **	31.8666 **	39.0597 ***
000635.SZ	12.1342 **	22.4461 ***	35.1346 ***	38.7178 ***
000639.SZ	21.8042 ***	41.4061 ***	49.9885 ***	62.4539 ***
000652.SZ	10.0423 *	22.7582 ***	24.5094 *	35.6892 **

Notes: *, ** and *** denotes statistical significance at the 10%, 5%, and 1% levels, respectively.

**Table 2:** The results of the GARCH test.

Stock Code	5-Lags	10-Lags	15-Lags	20-lags
000006.SZ	127.3155 ***	149.9881 ***	169.4464 ***	178.2135 ***
000009.SZ	206.7346 ***	219.8304 ***	221.5407 ***	224.5281 ***
000012.SZ	161.4745 ***	179.4592 ***	193.8611 ***	199.8437 ***
000014.SZ	231.9872 ***	238.4752 ***	243.2365 ***	250.4378 ***
000032.SZ	228.9215 ***	239.2971 ***	244.2169 ***	248.8801 ***
000055.SZ	163.0790 ***	183.5448 ***	186.8118 ***	189.9978 ***
000058.SZ	185.5711 ***	209.1581 ***	231.3122 ***	237.3947 ***
000060.SZ	181.5004 ***	189.1091 ***	221.4234 ***	226.1046 ***
000062.SZ	293.3655 ***	318.0177 ***	330.9509 ***	341.3959 ***
000063.SZ	144.8571 ***	156.4018 ***	163.2234 ***	170.6981 ***
000070.SZ	261.1783 ***	248.7214 ***	262.1619 ***	267.1721 ***
000088.SZ	199.2880 ***	219.7765 ***	234.2186 ***	241.0107 ***
000151.SZ	236.1484 ***	254.6980 ***	259.5269 ***	268.1101 ***
000417.SZ	254.2967 ***	275.6469 ***	285.6959 ***	309.9711 ***
000428.SZ	289.8030 ***	297.0388 ***	300.2933 ***	304.8884 ***
000488.SZ	209.8855 ***	227.1245 ***	234.4095 ***	239.7541 ***
000501.SZ	166.3387 ***	208.5034 ***	213.3474 ***	216.3341 ***
000506.SZ	178.4966 ***	186.1122 ***	189.4991 ***	194.8627 ***
000544.SZ	245.3693 ***	253.3165 ***	267.4441 ***	271.1274 ***
000545.SZ	118.8800 ***	131.7289 ***	149.6220 ***	168.4821 ***
000548.SZ	272.6703 ***	296.1373 ***	303.4039 ***	310.8151 ***
000551.SZ	232.2056 ***	241.4726 ***	245.8694 ***	245.7574 ***
000552.SZ	231.5626 ***	241.2432 ***	274.7245 ***	285.2567 ***
000554.SZ	208.7359 ***	223.8271 ***	231.5750 ***	233.5482 ***
000559.SZ	311.8441 ***	321.3317 ***	334.5868 ***	351.0071 ***
000561.SZ	268.0727 ***	277.8340 ***	286.8747 ***	292.8237 ***
000565.SZ	229.6917 ***	242.0975 ***	263.9275 ***	265.7864 ***
000572.SZ	179.4836 ***	195.8057 ***	200.9513 ***	205.6801 ***
000570.SZ	272.4294 ***	303.6636 ***	320.8354 ***	323.7563 ***
000576.SZ	254.1562 ***	264.0926 ***	279.6719 ***	281.7115 ***
000597.SZ	248.8782 ***	255.9566 ***	263.5923 ***	271.2491 ***
000600.SZ	214.9866 ***	218.9253 ***	239.0820 ***	242.9311 ***
000603.SZ	221.6153 ***	247.1927 ***	251.0414 ***	260.9005 ***
000619.SZ	338.7260 ***	345.4322 ***	369.5048 ***	369.2384 ***
000628.SZ	239.2112 ***	252.4262 ***	255.7817 ***	258.4453 ***
000629.SZ	51.1104 ***	51.9229 ***	57.4383 ***	65.2544 ***
000631.SZ	206.7708 ***	225.3945 ***	241.8617 ***	250.3248 ***
000635.SZ	135.8456 ***	139.1217 ***	142.8570 ***	142.3168 ***
000639.SZ	182.9437 ***	187.7796 ***	196.4492 ***	206.4381 ***
000652.SZ	176.4028 ***	206.0478 ***	214.2256 ***	213.9942 ***

Notes: *** denotes statistical significance at the 1% level.

**Table 3:** Contemporaneous correlations between daily return volatility and the logarithm value of Baidu search volume index (BSVI). This table represents the contemporaneous correlation coefficients between daily return volatility and the logBSVI. The daily return volatility was evaluated by GARCH(1,1), and the BSVI was downloaded from Baidu website (http://index.baidu.com/). “Pearson” denotes the Pearson correlation coefficients and “Spearman” denotes the Spearman correlation coefficients.

Stock Code	Pearson	Spearman	Stock Code	Pearson	Spearman
000006.SZ	0.6350 ***	0.6116 ***	000548.SZ	0.6844 ***	0.6774 ***
000009.SZ	0.5438 ***	0.5759 ***	000551.SZ	0.5720 ***	0.5755 ***
000012.SZ	0.5695 ***	0.6016 ***	000552.SZ	0.4721 ***	0.5402 ***
000014.SZ	0.7483 ***	0.7943 ***	000554.SZ	0.6373 ***	0.6269 ***
000032.SZ	0.6620 ***	0.5754 ***	000559.SZ	0.7577 ***	0.6282 ***
000055.SZ	0.5939 ***	0.6426 ***	000561.SZ	0.6496 ***	0.6368 ***
000058.SZ	0.6933 ***	0.6983 ***	000565.SZ	0.7033 ***	0.5856 ***
000060.SZ	0.7179 ***	0.7161 ***	000572.SZ	0.2658 ***	0.2312 ***
000062.SZ	0.7943 ***	0.7083 ***	000570.SZ	0.6686 ***	0.6296 ***
000063.SZ	0.7191 ***	0.7783 ***	000576.SZ	0.8218 ***	0.9019 ***
000070.SZ	0.7676 ***	0.6209 ***	000597.SZ	0.6126 ***	0.6302 ***
000088.SZ	0.5323 ***	0.5504 ***	000600.SZ	0.6494 ***	0.7218 ***
000151.SZ	0.7625 ***	0.6843 ***	000603.SZ	0.6619 ***	0.6735 ***
000417.SZ	0.6989 ***	0.7001 ***	000619.SZ	0.7267 ***	0.7798 ***
000428.SZ	0.6507 ***	0.5203 ***	000628.SZ	0.6563 ***	0.6311 ***
000488.SZ	0.5604 ***	0.5268 ***	000629.SZ	0.5891 ***	0.7649 ***
000501.SZ	0.5668 ***	0.5873 ***	000631.SZ	0.7011 ***	0.6506 ***
000506.SZ	0.7013 ***	0.7260 ***	000635.SZ	0.4228 ***	0.3719 ***
000544.SZ	0.6417 ***	0.6558 ***	000639.SZ	0.6920 ***	0.7639 ***
000545.SZ	0.6415 ***	0.6238 ***	000652.SZ	0.5663 ***	0.6709 ***

Notes: *** denotes statistical significance at the 1% levels.

**Table 4:** The mutual information between the daily return volatility and abnormal Baidu search volume. This table reports the mutual information between the daily return volatility and abnormal Baidu search volume. The daily return volatility is the GARCH(1,1) volatility of Bollerslev [16], and the abnormal Baidu search volume (AbSearch) is calculated by Model 3.

Stock Code	Mutual Information	Stock Code	Mutual Information
000006.SZ	0.7427	000548.SZ	0.6864
000009.SZ	0.7552	000551.SZ	0.6625
000012.SZ	0.5988	000552.SZ	0.7403
000014.SZ	0.6822	000554.SZ	0.7728
000032.SZ	0.7541	000559.SZ	0.7279
000055.SZ	0.6711	000561.SZ	0.6869
000058.SZ	0.7179	000565.SZ	0.6817
000060.SZ	0.7687	000572.SZ	0.8038
000062.SZ	0.7136	000570.SZ	0.7769
000063.SZ	0.7878	000576.SZ	0.6755
000070.SZ	0.6836	000597.SZ	0.6933
000088.SZ	0.6865	000600.SZ	0.6991
000151.SZ	0.7326	000603.SZ	0.7440
000417.SZ	0.7225	000619.SZ	0.7093
000428.SZ	0.7077	000628.SZ	0.6846
000488.SZ	0.7558	000629.SZ	0.6222
000501.SZ	0.7073	000631.SZ	0.7017
000506.SZ	0.7357	000635.SZ	0.7117
000544.SZ	0.6833	000639.SZ	0.6755
000545.SZ	0.6853	000652.SZ	0.6752

**Table 5:** Estimates of extended GARCH (1,1) model.

Stock Code	α	*t*-Value	β	*t*-Value	λ	*t*-Value	α + β
000006.SZ	0.3275 ***	6.76	0.4355 ***	8.58	1.4196 ***	7.08	0.7630
000009.SZ	0.2766 ***	7.31	0.4708 ***	11.45	2.5797 ***	8.77	0.7474
000012.SZ	0.4678 ***	9.39	0.1253 ***	2.81	0.6176 ***	9.51	0.5931
000014.SZ	0.4026 ***	7.23	0.1878 ***	3.93	1.1363 ***	11.29	0.5904
000032.SZ	0.4138 ***	6.00	0.3026 ***	3.98	1.4328 ***	6.77	0.7164
000055.SZ	0.5191 ***	9.01	0.0861 *	1.68	1.4104 ***	10.15	0.6052
000058.SZ	0.3105 ***	5.85	0.1790 ***	3.64	1.1872 ***	11.96	0.4895
000060.SZ	0.3959 ***	7.21	0.3349 ***	5.33	1.6069 ***	6.91	0.7308
000062.SZ	0.4127 ***	6.84	0.2809 ***	4.84	1.6888 ***	9.97	0.6936
000063.SZ	0.2923 ***	6.17	0.4882 ***	8.99	1.6272 ***	6.45	0.7805
000070.SZ	0.3485 ***	5.9	0.2478 ***	3.78	1.0664 ***	7.99	0.5963
000088.SZ	0.3229 ***	6.94	0.4895 ***	8.71	1.3952 ***	7.88	0.8124
000151.SZ	0.3279 ***	5.66	0.2568 ***	3.70	1.3112 ***	9.61	0.5847
000417.SZ	0.5668 ***	10.32	0.2694 ***	7.34	1.8860 ***	8.27	0.8362
000428.SZ	0.6182 ***	9.23	0.2467 ***	11.19	2.2509 ***	7.32	0.8649
000488.SZ	0.3507 ***	6.10	0.2504 ***	3.42	1.5869 ***	6.08	0.6011
000501.SZ	0.2791 ***	6.56	0.4980 ***	10.03	2.5483 ***	5.70	0.7771
000506.SZ	0.4896 ***	6.46	0.3344 ***	7.39	0.9747 ***	7.12	0.8240
000544.SZ	0.5815 ***	10.42	0.2937 ***	7.42	1.5176 ***	8.42	0.8752
000545.SZ	0.2750 ***	5.24	0.3463 ***	4.28	0.7839 ***	7.54	0.6213
000548.SZ	0.4858 ***	8.63	0.2981 ***	5.91	1.0613 ***	9.50	0.7839
000551.SZ	0.3274 ***	6.91	0.5260 ***	6.87	0.8253 ***	4.12	0.8534
000552.SZ	0.4378 ***	9.33	0.2068 ***	4.04	1.9028 ***	8.63	0.6446
000554.SZ	0.3346 ***	6.32	0.3040 ***	4.55	1.9121 ***	8.52	0.6386
000559.SZ	0.4647 ***	9.44	0.3338 ***	10.7	2.7574 ***	11.15	0.7985
000561.SZ	0.4894 ***	8.23	0.1391 **	2.72	1.2136 ***	8.36	0.6285
000565.SZ	0.4156 ***	6.23	0.2874 ***	4.69	1.0779 ***	8.71	0.7030
000572.SZ	0.2432 ***	5.88	0.2796 ***	3.71	1.0002 ***	10.64	0.5228
000570.SZ	0.4308 ***	8.62	0.4063 ***	8.92	3.4746 ***	7.05	0.8371
000576.SZ	0.7076 ***	10.99	0.1333 ***	5.40	1.1049 ***	16.31	0.8409
000597.SZ	0.4523 ***	7.68	0.1750 **	2.97	1.2753 ***	6.96	0.6273
000600.SZ	0.4189 ***	7.20	0.2645 ***	5.81	1.6152 ***	12.89	0.6834
000603.SZ	0.3137 ***	5.42	0.4218 ***	6.8	1.6865 ***	6.40	0.7355
000619.SZ	0.5090 ***	8.75	0.2387 ***	5.44	2.0084 ***	7.01	0.7477
000628.SZ	0.3317 ***	5.99	0.1746 **	2.72	1.1340 ***	8.64	0.5063
000629.SZ	0.3255 ***	4.43	0.3858 ***	4.42	0.6925 ***	5.59	0.7113
000631.SZ	0.4233 ***	8.51	0.2695 ***	5.93	1.6231 ***	11.47	0.6928
000635.SZ	0.3423 ***	5.70	0.1835 **	2.63	1.1718 ***	9.39	0.5258
000639.SZ	0.3883 ***	6.74	0.2836 ***	5.46	0.8062 ***	6.57	0.6719
000652.SZ	0.4641 ***	8.29	0.2115 ***	4.58	1.0943 ***	10.74	0.6756

Notes: *and *** denotes statistical significance at the 10% and 1% level, respectively.

**Table 6:** Improvement by the extended model.

α + β	Min	Max	Mean	SD
Basic GARCH (1,1)	0.7695	0.9985	0.9040	0.06697
Extended GARCH (1,1)	0.4895	0.8752	0.6983	0.1051
Basic GARCH (1,1)-Extended GARCH (1,1)	0.06535 ***	0.4309 ***	0.2057 ***	0.08369 ***

Notes: *** denotes statistical significance at the 1% level.
